# Two New Oleanane-Type Saponins with Anti-Proliferative Activity from *Camellia oleifera* Abel. Seed Cake

**DOI:** 10.3390/molecules21020188

**Published:** 2016-02-04

**Authors:** Jian-Fa Zong, Yun-Ru Peng, Guan-Hu Bao, Ru-Yan Hou, Xiao-Chun Wan

**Affiliations:** State Key Laboratory of Tea Plant Biology and Utilization, Anhui Agricultural University, Hefei 230036, China; zonjfa@sina.com (J.-F.Z.); pengyunru1101@sina.com (Y.-R.P.); baoguanhu@ahau.edu.cn (G.-H.B.)

**Keywords:** *Camellia* tea oil, saponin, oleiferasaponins, cytotoxic activity

## Abstract

Two new oleanane-type saponins, named oleiferasaponins C_4_ (**1**) and C_5_ (**2**), were isolated from *Camellia oleifera* Abel. seed cake residue. Their respective structures were identified as 16*α*-hydroxy-22α-*O*-angeloyl-23α-aldehyde-28-dihydroxymethylene-olean-12-ene-3β-*O*-[β-d-galacto-pyranosyl-(1→2)]-[β-d-glucopyranosyl-(1→2)-β-d-galactopyranosy-(1→3)]-β-d-glucopyranosid-uronic acid methyl ester (**1**) and 16*α*-hydroxy-22α-*O*-angeloyl-23α-aldehyde-28-dihydroxy-methylene-olean-12-ene-3β-*O*-[β-d-galactopyranosyl-(1→2)]-[β-d-galactopyranosyl-(1→3)]-β-d-glucopyranosiduronic acid methyl ester (**2**) through 1D- and 2D-NMR, HR-ESI-MS, and GC-MS spectroscopic methods. The two compounds exhibited potent cytotoxic activities against five human tumor cell lines (BEL-7402, BGC-823, MCF-7, HL-60 and KB).

## 1. Introduction

**Figure 1:**
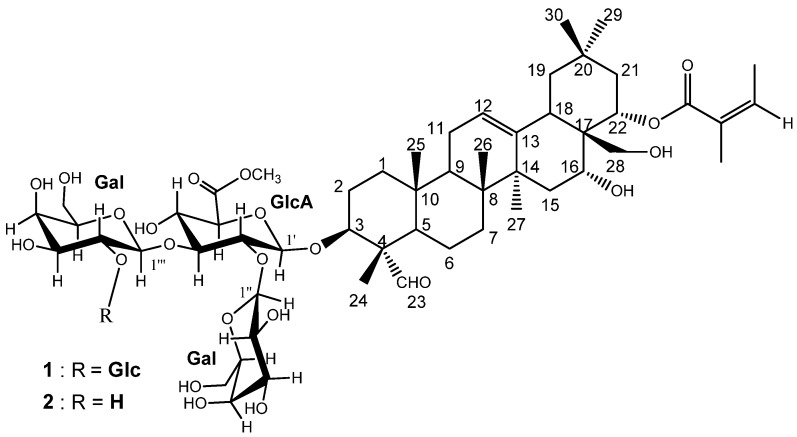
Structures of oleiferasaponins C_4_ and C_5_ (**1**, **2**).

*Camellia oleifera* Abel. seed saponins are triterpenoidic natural compounds that have potent pharmacological and biological activities, including antimicrobial [1,2,3], antioxidant [4], and gastroprotective [5] effects. *Camellia* saponins are commercially used as biosurfactants [6], biopesticides [7] and detergents [8]. New saponin monomers continue to be isolated and identified from the seed cake of *Camellia oleifera*, with many showing potent anti-proliferative activity against human cancer cell lines [9,10,11]. *C. oleifera* seed cake, is an agricultural byproduct resulting from the extraction of the edible tea oil from the seeds. The seed cake contains approximately 10% saponins by weight, a fraction comprised of more than 30 types of saponins [3,12]. There are 10 saponins [1,9,10,12,13,14] that have been recently identified in tea seed cake, including nine previously unreported from any source. Seven of the saponins have been reported to have anti-proliferative activity against human cancer cell lines. A previous study indicated that different tea saponin structures display different anti-tumor activity [15]. Some of the varied saponins in *C. oleifera* seed cake are more difficult to isolate and have complex chemical structures that are difficult to identify, hindering the study of their structure and function. In order to study the saponin structure-activity relationships, it is necessary to isolate and identify additional monomer compounds from the crude saponin fraction, especially triterpene saponins. Identification of the functions of oleanane-type saponins in *C. oleifera* and clear illustration of structure-activity relationship would increase the effective utilization of tea cake, particularly in pharmaceutical applications. As a part of our ongoing study of the constituents of the *C. oleifera* seed cake of, we recently isolated two new oleanane-type saponins. We report herein the isolation and structural elucidation of the new saponins, namely oleiferasaponins C_4_ and C_5_ (Figure 1), along with their anti-proliferative activity against five human tumor cell lines, namely BEL-7402, BGC-823, MCF-7, HL-60 and KB.

## 2. Results and Discussion

### 2.1. Isolation and Characterization of the Triterpenoid Saponins

The *n*-BuOH fraction obtained from the 70% EtOH extract of defatted seed cake of *C. oleifera* was further separated through silica gel column and gel permeation chromatography on Sephadex LH-20, and by repeated reversed-phase C_18_ column chromatography. Two new oleanane-type saponins were thus obtained. Their structures were determined mainly by 600 MHz NMR experiments and high resolution electro-spray ionization mass spectrometry (HR-ESI-MS).

**Figure 2:**
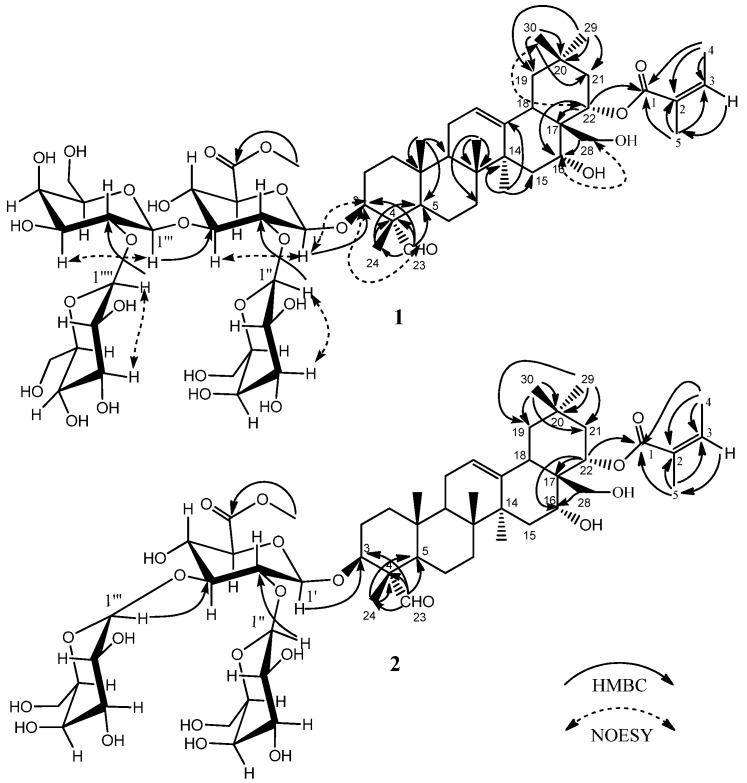
Key HMBC and NOESY correlations within oleiferasaponins C_4_ and C_5_ (**1**, **2**).

Compound **1** was separated as a white amorphous powder. The molecular formula C_60_H_9__4_O_2__7_ was deduced from the HR-ESI-MS [M + Na]^+^ ion at *m*/*z* 1269.5875. The IR spectrum (cm^−1^) showed the presence of hydroxyl (broad peak around 3416), carbonyl (1719), olefinic (1640), and ether (1078, 1044) functional groups. The ^13^C-NMR spectrum (Table 1) displayed the resonances of 60 carbons, ascribable to nine methyls, twelve methylenes, twenty-nine methines, and ten quaternary carbons as revealed by the HSQC experiment. Of the 60 carbons, 30 were assigned to the triterpene moiety. The ^1^H-NMR spectrum (Table 1) showed six methyl groups at *δ* 0.78, 0.81, 1.05, 1.29,1.42, and 1.80 (3H, each, all s, H_3_-25, 26, 29, 30, 24, 27), one methylene group at *δ* 3.54, 3.66 (2H, both m, H_2_-28), three methine protons bearing oxygens at *δ* 4.02 (1H, m, H-3), 4.60 (1H, br s, H-16), and 6.22 (1H, dd, *J* = 12.0, 6.0 Hz, H-22), an aldehyde signal at *δ* 9.91 (1H, s, H-23), and one olefinic proton signal at *δ* 5.35 (1H, br s, H-12), which indicated an oleanane aglycone. Furthermore, the signals of angeloyl (Ang) group at [*δ* 5.92 (1H, dq-like, 22-*O*-Ang-3), 2.09 (3H, d, *J* = 7.2 Hz, 22-*O*-Ang-4), 1.95 (3H, s, 22-*O*-Ang-5) were observed. The location of the Ang group at C-22 was confirmed by the HMBC experiment (Figure 2). In addition to these, the anomeric proton signals at *δ*_H_ 4.80 (1H, d, *J* = 7.2 Hz, H-1′ of glucuronic acid), 5.13 (1H, d, *J* = 7.8 Hz, H-1′′′′ of glucose), 5.75 (1H, d, *J* = 7.2 Hz, H-1′′′ of galactose), and 5.79 (1H, d, *J* = 7.8 Hz, H-1′′ of galactose), which showed the HSQC correlation to *δ*_C_ 103.9 (C-1′ of glucuronic acid), 106.9 (C-1′′′′ of glucose), 101.6 (C-1′′′ of galactose), and 103.1 (C-1′′ of galactose), respectively, indicated the presence of four sugar residues (Table 1). The position of the sugar components was determined on the basis of the HMBC experiment (Figure 2), which showed long-range correlations between the following proton and carbon pairs: GlcA-H-1′ and *δ*_C_ 84.1 (C-3 of the aglycone), indicating that the glycosidic chain was located at C-3 of the aglycone, Gal-H-1′′ and *δ*_C_ 78.4 (GlcA-C-2′), Gal-H-1′′′ and *δ*_C_ 83.9 (GlcA-C-3′), and Glc-H-1′′′′ and *δ*_C_ 83.5 (Gal-C-2′). Moreover, the signals at *δ*_C_ 52.1 and *δ*_H_ 3.69 were identified as those of one OCH_3_ group located at C-6′ of the glucuronic acid, which was supported by the HMBC correlation between *δ*_C_ 170.0 (GlcA-C-6′) and *δ*_H_ 3.69 (OCH_3_) (Figure 2). These data from compound **1** were further confirmed by acid hydrolysis and GC-MS analysis, which revealed one unit of d-glucuronic acid methyl ester, two units of d-galactose, and one unit of d-glucose. The relative configuration of **1** was established from its NOESY spectrum (Figure 2). The cross peaks between H-22 at *δ*_H_ 6.22 and H-30 at *δ*_H_ 1.29, as well as those between H-16 at *δ*_H_ 4.60 and H-28 at *δ*_H_ 3.54, 3.66, suggested that H-22 and H-16 are both β-oriented, which means that the Ang group at C-22 and 16-OH group are both α-orientations. The H-3 at *δ*_H_ 4.02 correlated with H-23 at *δ*_H_ 9.91, indicating that the glycosidic chain group at C-3 is β-configured. Thus, the structure of 1 was elucidated to be 16α-hydroxy-22α-*O*-angeloyl-23α-aldehyde-28-dihydroxy-methyleneolean-12-ene-3β-*O*-[β-d-galactopyranosyl-(1→2)]-[β-d-glucopyranosyl-(1→2)-β-d-galacto- pyranosy-(1→3)]-β-d-glucopyranosiduronic acid methyl ester, which is a new compound that we named oleiferasaponin C_4_.

**Table 1:** ^1^H- and ^13^C-NMR spectroscopic data of oleiferasaponins C_4_ and C_5_ in C_5_D_5_N.

Position	Oleiferasaponin C_4_ (1)		Oleiferasaponin C_5_ (2)	
	*δ*_C_	*δ*_H_	*δ*_C_	*δ*_H_
1	38.0	0.87 m, 1.37 m	38.0	0.90 m, 1.41 m
2	25.1	1.78 m, 2.05 m	25.1	1.83 m, 2.05 m
3	84.1	4.02 m	84.5	4.04 m
4	55.0		55.0	
5	48.3	1.37 m	48.5	1.37 m
6	20.3	0.90 m, 1.35 m	20.3	0.96 m, 1.35 m
7	32.2	1.11 m, 1.51 m	32.3	1.13 m, 1.54 m
8	40.2		40.2	
9	46.7	1.75 m	46.7	1.78 m
10	35.9		36.0	
11	23.7	1.76 m, 1.84 m	23.7	1.77 m, 1.86 m
12	122.9	5.35 br s	122.9	5.36 br s
13	143.7		143.7	
14	41.6		41.7	
15	35.0	1.49 m, 1.83 m	35.0	1.50 m, 1.85 m
16	69.9	4.60 br s	70.0	4.61 br s
17	44.7		44.8	
18	40.8	3.05 m	40.8	3.04 m
19	47.3	1.31 m, 2.88 m	47.4	1.32 m, 2.89 m
20	32.0		32.0	
21	41.5	2.05 m, 2.83 m	41.6	2.05 m, 2.83 m
22	72.8	6.22 dd (6.0, 12.0)	72.9	6.23 dd (6.0, 12.0)
23	209.9	9.91 s	210.0	9.93 s
24	11.0	1.42 s	11.0	1.46 s
25	15.7	0.78 s	15.7	0.80 s
26	16.7	0.81 s	17.0	0.82 s
27	27.5	1.80 s	27.5	1.81 s
28	63.4	3.54 m, 3.66 m	63.5	3.56 m, 3.66 m
29	33.4	1.05 s	33.4	1.05 s
30	25.2	1.29 s	25.2	1.29 s
22-*O*-Ang				
Ang-1	167.9		167.9	
Ang-2	129.4		129.4	
Ang-3	136.6	5.92 (dq-like)	136.5	5.92 (dq-like)
Ang-4	15.8	2.09 d (7.2)	15.8	2.09 d (6.6)
Ang-5	20.9	1.95 s	20.9	1.96 s
3-*O*-				
GlcA-1′	103.9	4.80d (7.2)	103.7	4.83 d (7.2)
GlcA-2′	78.4	4.61 m	78.1	4.25 m
GlcA-3′	83.9	4.36 m	87.4	4.17 m
GlcA-4′	70.4	4.37 m	71.3	4.30 m
GlcA-5′	76.9	4.11 m	76.2	4.36 m
GlcA-6′	170.0		169.8	
COOMe	52.1	3.69s	52.1	3.69 s
Gal-1′′	103.1	5.79 d (7.8)	104.3	5.50 d (7.8)
Gal-2′′	73.6	4.50 m	73.6	4.46 m
Gal-3′′	74.8	4.33 m	75.3	4.11 m
Gal-4′′	70.3	4.47 m	70.0	4.44 m
Gal-5′′	76.3	4.23 m	76.7	3.96 m
Gal-6′′	61.7	4.00 m, 4.35 m	61.9	4.45 m, 4.53 m
Gal-1′′′	101.6	5.75 d (7.2)	105.1	5.19 d (7.2)
Gal-2′′′	83.5	4.55 m	72.9	4.49 m
Gal-3′′′	75.1	4.31 m	75.5	4.12 m
Gal-4′′′	69.6	4.51 m	70.1	4.57 m
Gal-5′′′	76.5	4.46 m	77.3	4.10 m
Gal-6′′′	62.2	4.50 m, 4.55 m	61.9	4.31 m, 4.35 m
Glc-1′′′′	106.9	5.13 d (7.8)		
Glc-2′′′′	76.3	4.28 m		
Glc-3′′′′	78.5	3.74 m		
Glc-4′′′′	71.2	4.36 m		
Glc-5′′′′	78.3	4.12 m		
Glc-6′′′′	62.4	4.39 (2H, m)		

^1^H(*δ* ppm, *J* Hz, s: singlet; d: doublet; brs: broad singlet; m: multiplet). Ang: angeloyl; GlcA: glucuronic acid; Gal: galactose; Glc: glucose.

Compound **2** was separated as a white amorphous powder. The molecular formula C_54_H_84_O_2__2_ was deduced from the HR-ESI-MS [M + Na]^+^ ion at *m*/*z* 1107.5358. The IR spectrum (cm^−1^) showed the presence of hydroxyl (broad peak around 3416), carbonyl (1739), olefinic (1641), and ether (1077, 1045) functional groups. The NMR (Table 1) data of **2** were similar to those of **1**, except for the absence of the glucose sugar unit at C-2′′′ of the galactose moiety. This means that there are only three sugar units in the glycosidic chain of compound **2** (Table 1). The anomeric proton signals were at *δ*_H_ 4.83 (1H, d, *J* = 7.2 Hz, H-1′ of glucuronic acid), 5.19 (1H, d, *J* = 7.2 Hz, H-1′′′ of glucose), and 5.50 (1H, d, *J* = 7.8 Hz, H-1′′ of galactose) and showed the HSQC correlation to *δ*_C_ 103.7 (C-1′ of glucuronic acid), 105.1 (C-1′′′ of galactose), and 104.3 (C-1′′ of galactose), respectively. The HMBC (Figure 2) correlations were between GlcA-H-1′ and *δ*_C_ 84.5 (C-3 of the aglycone), Gal-H-1′′ and *δ*_C_ 78.1 (GlcA-C-2′), and Gal-H-1′′′ and *δ*_C_ 87.4 (GlcA-C-3′). In addition, the data of **2** was further confirmed by acid hydrolysis and GC-MS analysis, which revealed one unit of d-glucuronic acid methyl ester and two units of d-galactose. Consequently, the structure of **2** was determined to be 16α-hydroxy-22α-*O*-angeloyl-23α-aldehyde-28-dihydroxymethylene-olean-12-ene-3β-*O*-[β-d-galactopyranosyl-(1→2)]-[β-d-galactopyranosyl-(1→3)]-β-d-glucopyranosiduronic acid methyl ester, also a new compound that we named oleiferasaponin C_5_.

### 2.2. Anti-Proliferative Assay on Human Tumor Cells

The isolated Compounds **1**, **2** and total seed cake saponins (*C. oleifera*) were tested against five human tumor cell lines (human hepatocellular carcinoma cells BEL-7402, human gastric carcinoma cells BGC-823, human breast cancer cells MCF-7, human promyelocytic leukemia cells HL-60 and human oral epidermoid carcinoma cells KB) using the MTT *in vitro* assay. Taxol, an anticancer agent, was used as the positive control. Both oleanane-type saponins exhibited effective anti-proliferative activity against the human tumor cell lines tested, especially compound **2**. Compound **2** showed potent anti-proliferative activities, with IC_50_ values ranging from 1.8 to 5.5 μM, while compound **1** displayed anti-proliferative activity with IC_50_ values between 6.5 and 15.1 μM. Total saponins showed anti-proliferative activity with IC_50_ values between 6.0 and 19.8 μM (Table 2).

**Table 2:** Anti-proliferative activity for compounds **1**, **2** and total saponins of *C. oleifera* against five human tumor cell lines.

Compound	Cell Lines IC_50_				
	BEL-7402	BGC-823	MCF-7	HL-60	KB
**1** (μM)	10.385	11.242	15.094	6.489	12.302
**2** (μM)	4.218	5.505	3.915	1.797	3.468
Total Saponins (μg/mL)	11.023	5.981	10.611	12.546	19.761

Taxol was used as a positive control.

The structure-activity relationships were inferred by comparison of the structure and the anti-proliferative activities on five human tumor cell lines. Compounds **1** and **2** have the same aglycone, but compound **2** lacks the glucose sugar moiety at position Gal-C-2′′′. The higher anti-proliferative activities of **2** compared with those of **1** on all of the tested tumor cell lines suggested that three saccharide units (rather than four saccharide units) leads to an increase in the biological potency. The results are consistent with Mu *et al.* [15]. In comparison with those of previously reported compounds [10], it seems that the Ang group at position C-22 and the free hydroxy group at C-28 in compound **2** may also play important roles in cytotoxicity. These results further support the idea that the anti-proliferative activity of saponins depends not only on isolated structural factors but also on combinatorial properties of both the aglycone and the sugar moieties.

## 3. Materials and Methods 

### 3.1. General Information

The following spectroscopic instruments were used to obtain physical data on the two new isolated saponins: ^1^H- and ^13^C-NMR, ^1^H-^1^H COSY, HSQC, HMBC and NOESY spectra were recorded in C_5_D_5_N using a DD2 (600 MHz) spectrometer (Agilent, Palo Alto, CA, USA) operating at 600 MHz for ^1^H and 150 MHz for ^13^C. Coupling constants were expressed in Hz and chemical shifts were given on a *δ* (ppm) scale with tetramethylsilane (TMS) as an internal standard. HR-ESI-MS spectra were determined on an Electrostatic Field Orbital Trap Mass Spectrometer (Thermo Scientific, Bremen, Germany) using an ESI source. IR was measured on a Nicolet 8700 FT-IR spectrophotometer (Thermo Scientific Instrument Co., Boston, MA, USA). The HPLC analyses were performed with Agilent Zorbax Eclipse Plus C_18_ HPLC column (250 mm × 4.6 mm i.d., 5 μm, Agilent Corp., Palo Alto, CA, USA) on an HPLC system composed of an LC-20AD pump with an SPD-M20A detector (Shimadzu Corp., Kyoto, Japan). The HPLC purifications were performed with a YMC-Pack ODS-A semi-preparative HPLC column (250 mm × 10 mm i.d., 5 μm, YMC Corp., Ltd., Kyoto, Japan) on a Varian Prostar HPLC instrument (Model 325) (Varian, Mulgrave, Australia) and an Agilent Zorbax Eclipse Plus C_18_ HPLC column on a Waters 2695 separation module combined with a Waters 2489 UV detector (Milford, MA, USA) with the detection wavelengths at 210 and 280 nm. GC-MS analyses were conducted on a GCMS-QP2010S (Shimadzu Corp., Kyoto, Japan) with DB-5MS column (i.d. = 0.25 μm, length = 30 m, Agilent Technologies). Column chromatography resins were silica gel (100–200 mesh, Qingdao Marine Chemical Co. Ltd., Qingdao, China) and Sephadex LH-20 (25–100 μm, Pharmacia Fine Chemical Co. Ltd., Uppsala, Sweden). Thin-layer chromatography (TLC) used precoated silica gel GF254 plates (Qingdao Marine Chemical Co. Ltd., Qingdao, China) and detection by spraying with 5% vanillin in 10% H_2_SO_4_-EtOH solution, followed by heating.

### 3.2. Plant Materials

Tea seed cakes of *C. oleifera* were obtained from a commercial tea oil producer in the city of Huangshan in Anhui Province, China. The samples had been defatted according to Lee *et al.* [16].

### 3.3. Extraction and Isolation

The tea seed cake (2.0 kg) was crushed into powder and extracted three times with 70% EtOH (3 × 10 L) at 60 °C under reflux each for 4 h. The extract was subjected to reduced pressure evaporation to obtain a concentrated EtOH solution (350 g). The concentrated solution was extracted successively with petroleum ether, EtOAc and *n*-BuOH. The *n*-BuOH fraction (80 g) was subjected to silica gel (100–200 mesh, 2.0 kg) column chromatography (70 mm × 600 mm) and eluted with stepwise gradients of EtOAc and MeOH (100:0, 90:10, 80:20, 70:30, 60:40, 50:50, 40:60, 30:70, 20:80, 10:90, 0:100, each 8.0 L). Each fraction was collected and analyzed by TLC. Fractions with similar R_f_ values were combined, yielding eight major fractions (A–H). Fraction G (6.5 g) was submitted to gel permeation chromatography on Sephadex LH-20 (50 mm × 400 mm) in MeOH to remove the pigments and flavones and yielded four major fractions (I-IV). Fraction II (2.0 g) was further subjected to semi-preparative HPLC (YMC-Pack ODS-A, 250 mm × 10 mm i.d., 5 μm, CH_3_CN-0.5% aqueous HCOOH (40:60, *v*/*v*), 2 mL/min) to yield five fractions [Fr. 1 (0.83 g), Fr. 2 (0.12 g), Fr. 3 (0.06 g), Fr. 4 (0.11 g), and Fr. 5 (0.55 g)]. Fractions 2 and 3 were further purified by HPLC [Agilent C_18_, 250 mm× 4.6 mm i.d., 5 μm, CH_3_CN-0.5% aqueous HCOOH (42:58, *v*/*v*), 1 mL/min] to afford Compounds **1** (8.0 mg) and 2 (5.2 mg).

### 3.4. Spectroscopic Data

Oleiferasaponin C_4_ (**1**): white amorphous powder; UV (MeOH) *λ*_max_ nm (log *ε*): 239 (4.16), 278 (4.28); IR (KBr) *ν*_max_ (cm^−1^): 3416, 2951, 2927, 1740, 1719, 1640, 1441, 1385, 1242, 1160, 1078, 1044; ^1^H-NMR (pyridine-*d*_5_) and ^13^C-NMR (pyridine-*d*_5_) spectroscopic data, see Table 1; HR-ESI-MS (positive ion mode): *m*/*z* 1269.5875 [M + Na]^+^ (calcd for C_60_H_9__4_O_2__7_ Na, 1269.5880), the data was shown in supplementary materials.

Oleiferasaponin C_5_ (**2**): white amorphous powder; UV (MeOH) *λ*_max_ nm (log *ε*): 209 (3.91), 280 (4.27); IR (KBr) *ν*_max_ (cm^−1^): 3416, 2927, 1739, 1720, 1641, 1442, 1385, 1242, 1162, 1077, 1045; ^1^H-NMR (pyridine-*d*_5_) and ^13^C-NMR (pyridine-*d*_5_) spectroscopic data, see Table 1; HR-ESI-MS (positive ion mode): *m*/*z* 1107.5356 [M + Na]^+^ (calcd for C_5__4_H_84_O_2__2_ Na, 1107.5352), the data was shown in supplementary materials.

### 3.5. Acid hydrolysis and GC-MS Analysis of the Sugar Moieties in **1** and **2**

Each saponin (0.8 mg) was dissolved in 1 M HCl (1 mL) for 3 h at 90 °C. The reaction mixture was extracted with chloroform, and the supernatant was evaporated to dryness under N_2_ flow. The residue was dissolved in 0.2 mL of pyridine containing l-cysteine methyl ester hydrochloride (10 mg/mL) and reacted at 70 °C for 1 h. This reaction was evaporated under N_2_ flow, after which 0.2 mL trimethylsilylimidazole (Adamas Reagent Co., Ltd, Shanghai, China) was added. The mixture was heated at 70 °C for another 1 h, and then partitioned between *n*-hexane and water. The organic phase was analyzed by GC-MS. Temperature conditions were as follows: injector temperature at 280 °C; the initial oven temperature was 160 °C for 1 min, then linearly increased to 200 °C at 6 °C/min. A further linear increase at 3 °C/min was performed to 280 °C, which was held for 5 min. The standard sugar samples were subjected to the same reaction and GC-MS conditions. The sugar units of compounds **1** and **2** were identified by comparison with authentic samples: d-xylose (t_R_ 16.93 min), d-glucose (t_R_ 21.67 min), d-galactose (t_R_ 22.31 min), d-glucuronic acid methyl ester (t_R_ 23.34 min). d-glucose, d-galactose and d-glucuronic acid methyl ester were identified in a ratio of 1:2:1 for compound **1**, d-galactose and d-glucuronic acid methyl ester were identified in a ratio of 2:1 for **2**.

### 3.6. Anti-Proliferative Activity Assay in Vitro

The procedure for the anti-proliferative activity assay was performed according to the MTT reduction method using the human tumor cell lines BEL-7402, BGC-823, MCF-7, HL-60 and KB (Nanjing KeyGEN BioTECH Co., LTD, Jiangsu, China). In brief, the human tumor cell lines in culture medium (100 μL) were placed in a cell of a 96-well plate at a concentration of 4 × 10^3^ cells/mL and incubated at 37 °C in 5% CO_2_ for 24 h. After 24 h, an additional 100 μL of complete medium with either: no additions (negative control), 0.1% DMSO (solvent control), 10 μg/mL Taxol (positive control), or different concentrations (0.391, 0.781, 1.562, 3.125, 6.25, 12.5, 25 and 50 μM) of 1 or 2 or total saponins (0.391, 0.781, 1.562, 3.125, 6.25, 12.5, 25 and 50 μg/mL). The treated cells were incubated as above for 72 h. Then, 20 μL of MTT solution (5 mg/mL) were added to the culture medium, and the reaction mixture was incubated as above for 4 h. After 4 h, the medium was discarded and 150 μL DMSO were added. The optical density (OD) of each well was measured at 490 nm using a Tunable Microplate Reader (EL-x800, BioTek Instruments, Winooski, VT, USA). The results were expressed as concentrations of compound producing 50% toxicity (IC_50_ value).

## 4. Conclusions

Two new oleanane-type saponins, namely oleiferasaponins C_4_ and C_5_ (**1**, **2**), were isolated from the seed cake of *Camellia oleifera* Abel. and identified. The anti-proliferative activity of the two compounds were investigated on five human tumor cell lines (BEL-7402, BGC-823, MCF-7, HL-60 and KB) and exhibited potent cytotoxic activities, especially compound **2**.

## Supplementary Materials

Supplementary materials can be accessed at: http://www.mdpi.com/1420-3049/21/2/188/s1. HR-ESR-MS and NMR spectra data of compounds **1** and **2** as supporting information.
