# Supplementary material for: Two New Oleanane-Type Saponins with Anti-Proliferative Activity from Camellia oleifera Abel. Seed Cake
Source: Molecules. 2016 Feb 4;21(2):188. doi: 10.3390/molecules21020188 (PMC6273491; doi:10.3390/molecules21020188)
Supplement: Supplementary file 1 [file molecules-21-00188-s001.pdf]

Supplementary Materials: Two New Oleanane-Type Saponins with Anti-Proliferative Activity from *Camellia oleifera* Abel. Seed Cake

Jian-Fa Zong, Yun-Ru Peng, Guan-Hu Bao, Ru-Yan Hou * and Xiao-Chun Wan *

Contents

Contents S1

Figure S1. Positive ion mode HR-ESI-MS spectrum of compound **1** S2

Figure S2. Positive ion mode HR-ESI-MS spectrum of compound **2** S2

Figure S3. ^{1}H-NMR spectrum of compound **1** S3

Figure S4. ^{13}C-NMR spectrum of compound **1** S3

Figure S5. ^{1}H-^{1}H COSY spectrum of compound **1** S4

Figure S6. HSQC spectrum of compound **1** S4

Figure S7. HMBC spectrum of compound **1** S5

Figure S8. NOESY spectrum of compound **1** S5

Figure S9. ^{1}H-NMR spectrum of compound **2** S6

Figure S10. ^{13}C-NMR spectrum of compound **2** S6

Figure S11. ^{1}H-^{1}H COSY spectrum of compound **2** S7

Figure S12. HSQC spectrum of compound **2** S7

Figure S13. HMBC spectrum of compound **2** S8

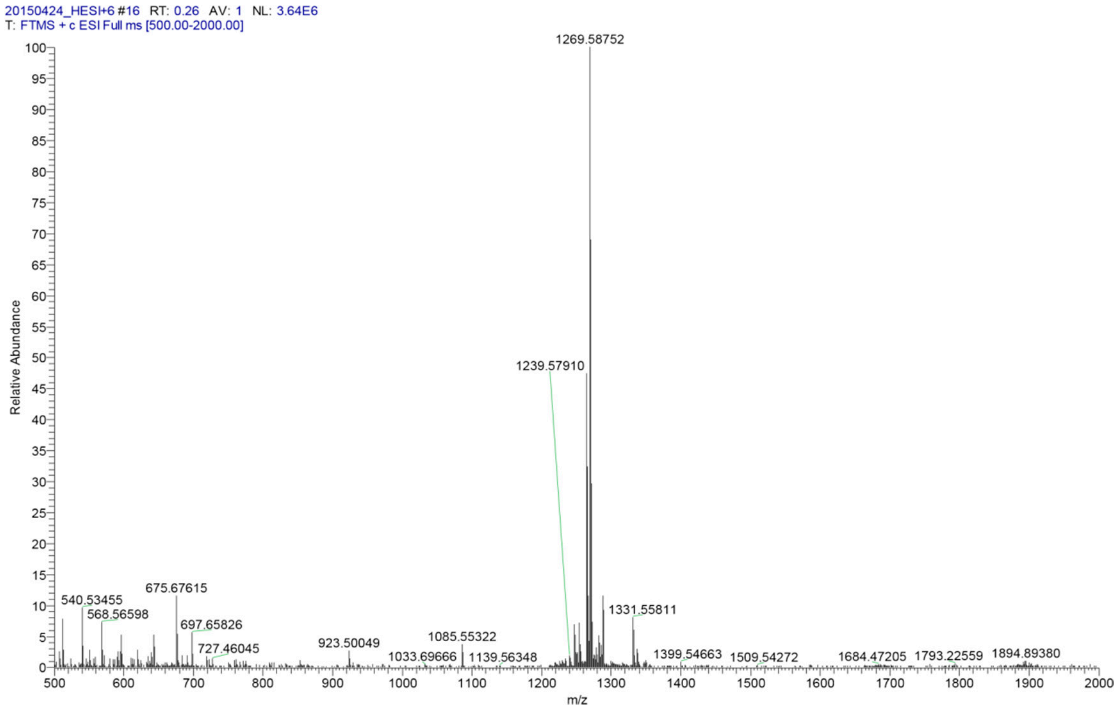

Figure S1. Positive ion mode HR-ESI-MS spectrum of compound **1**.

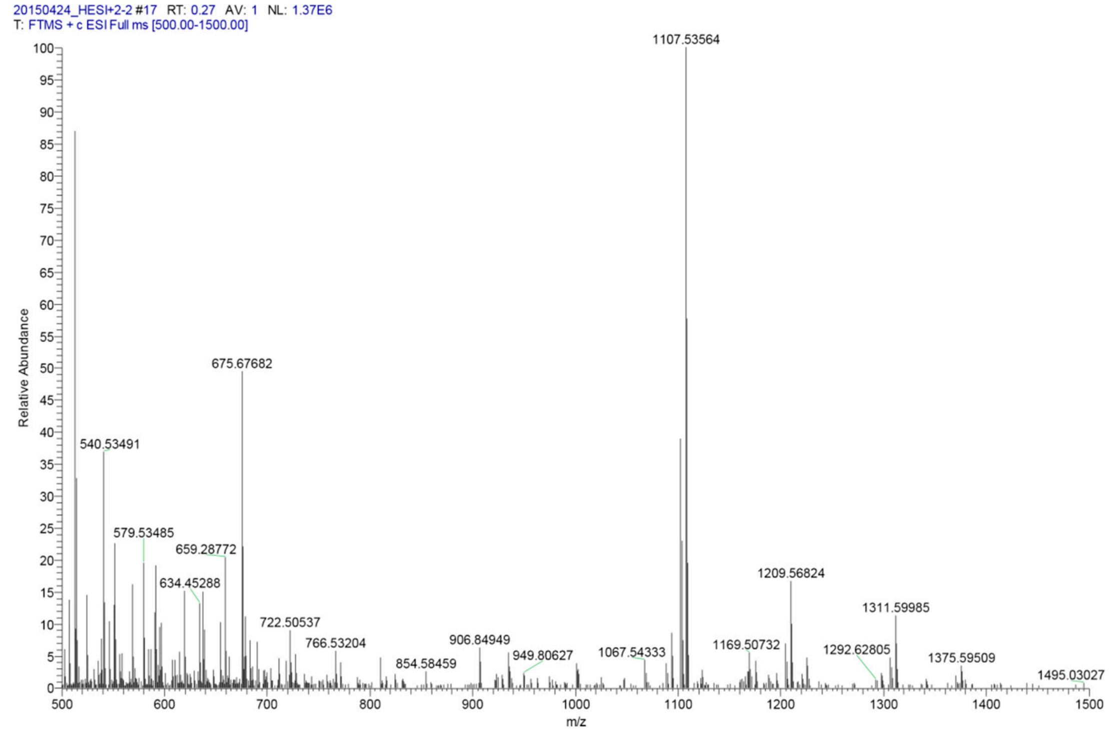

Figure S2. Positive ion mode HR-ESI-MS spectrum of compound **2**.

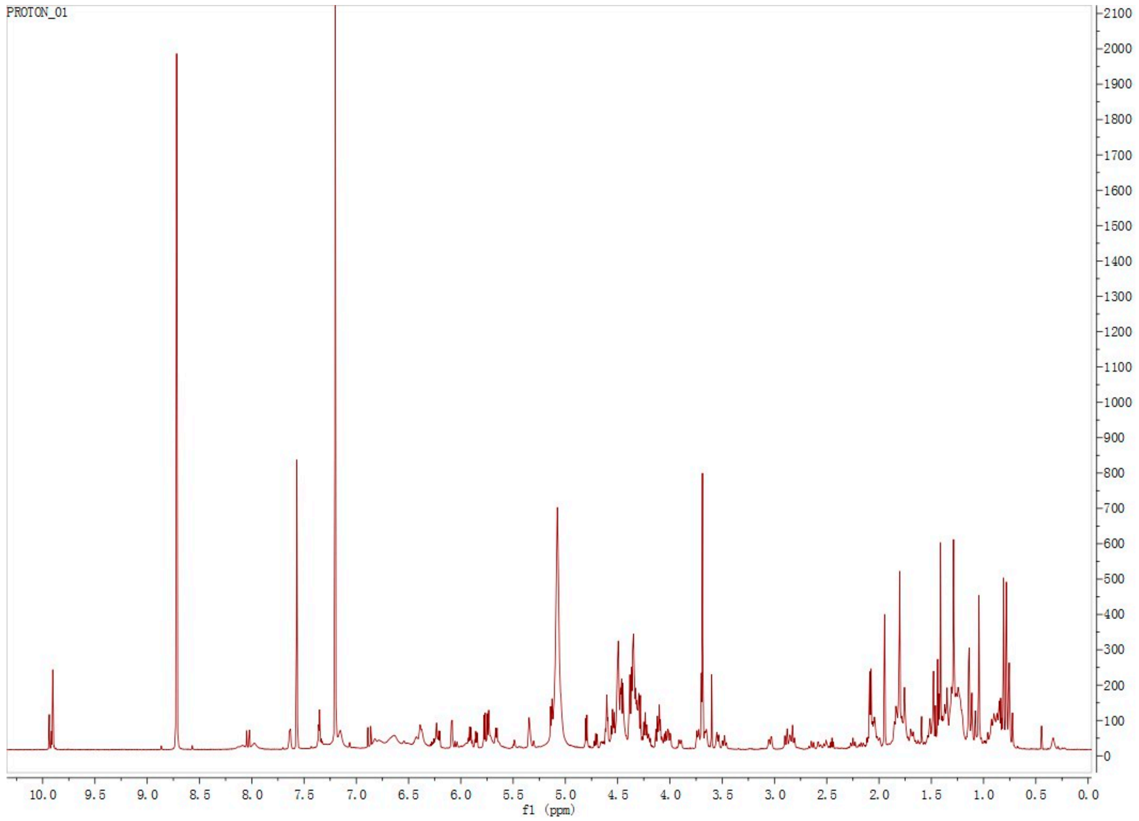

Figure S3. ^{1}H-NMR spectrum of compound **1**.

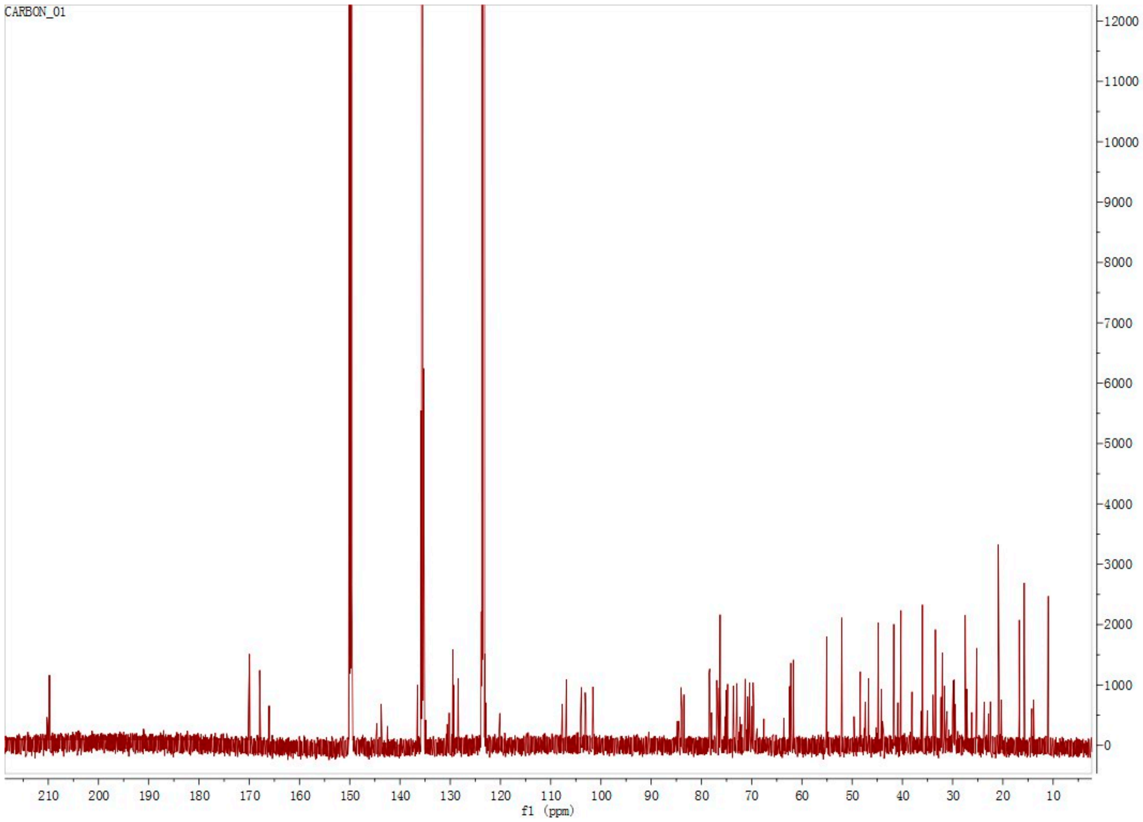

Figure S4. ^{13}C-NMR spectrum of compound **1**.

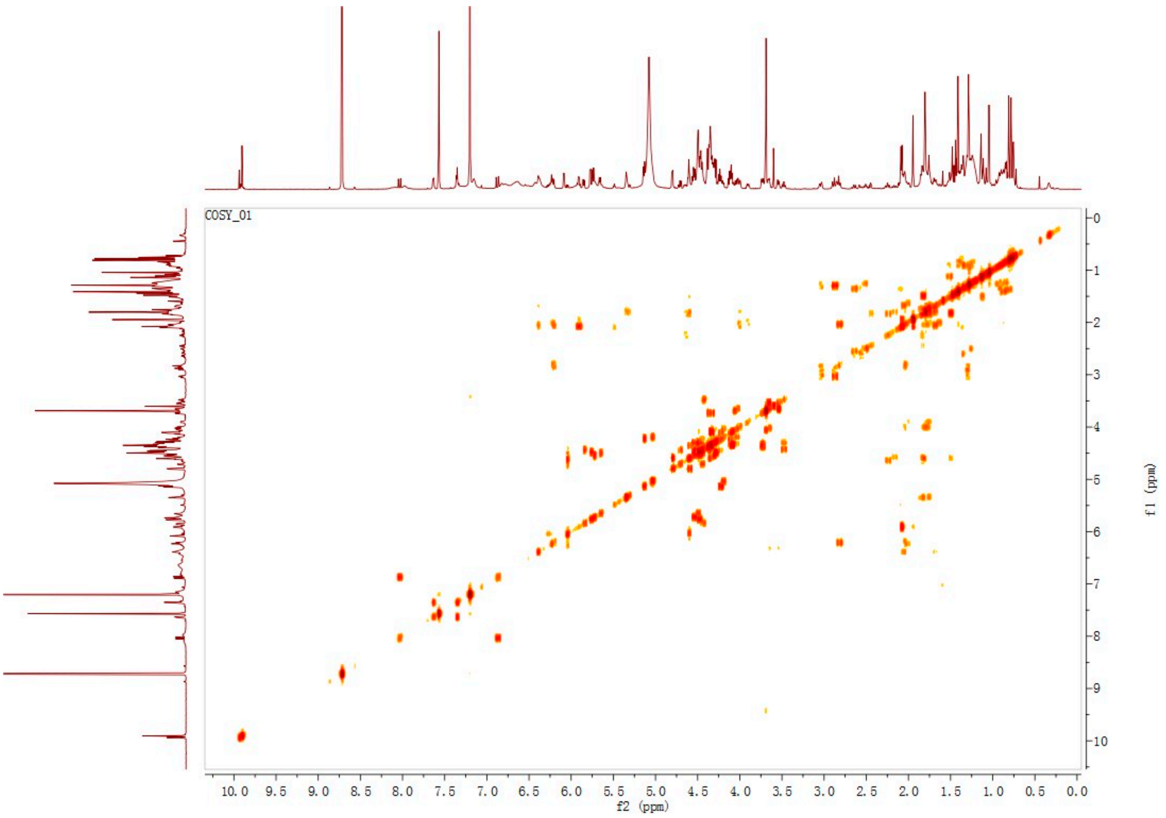

Figure S5. ^{1}H-^{1}H COSY spectrum of compound **1**.

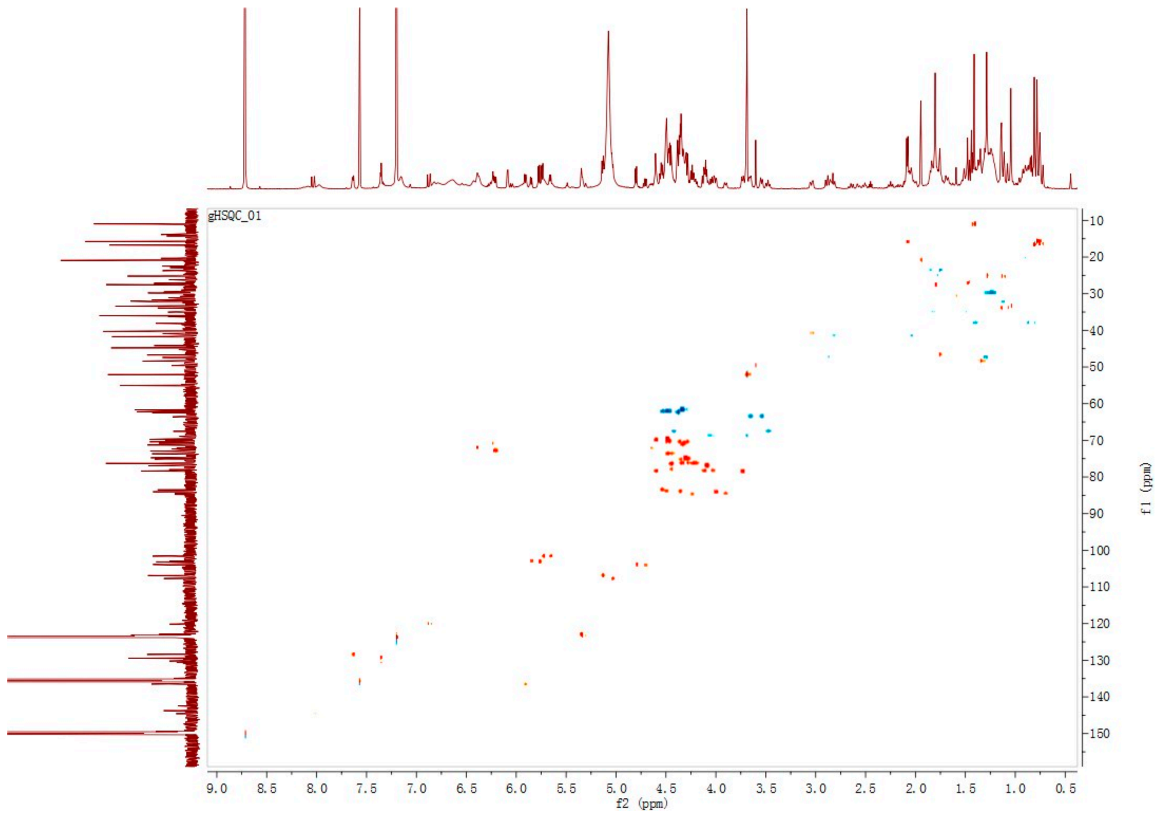

Figure S6. HSQC spectrum of compound **1**.

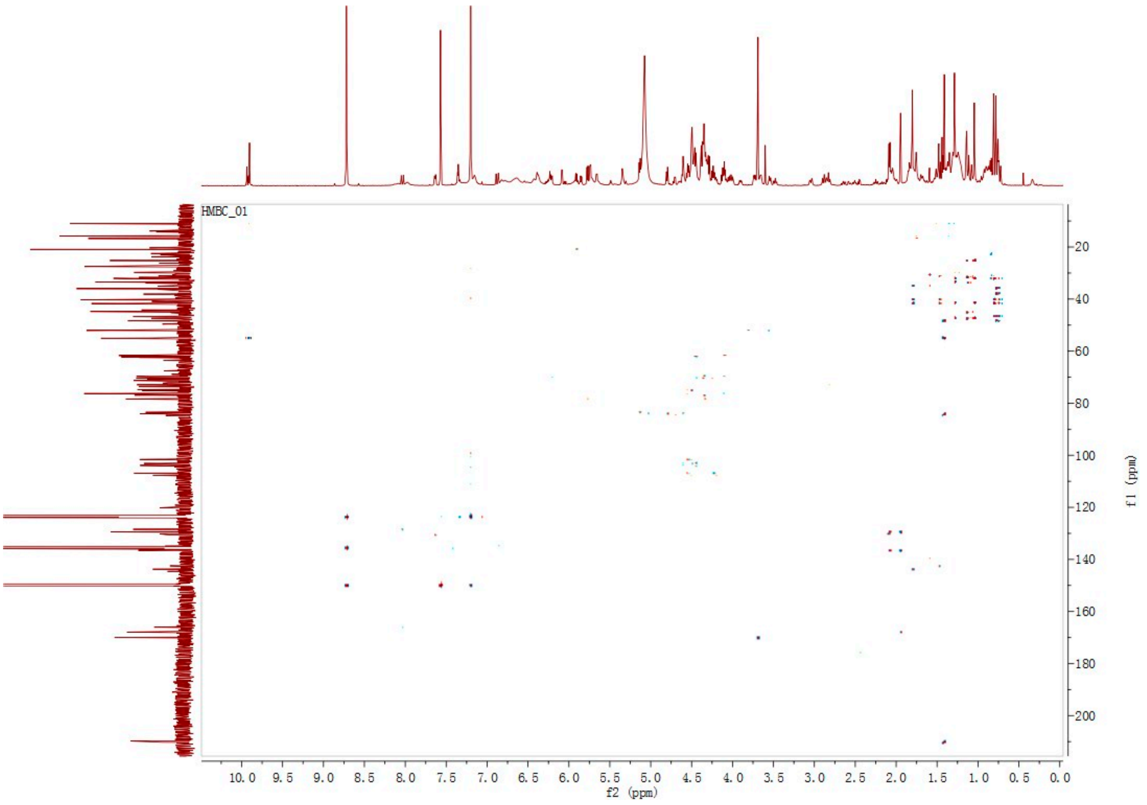

Figure S7. HMBC spectrum of compound **1**.

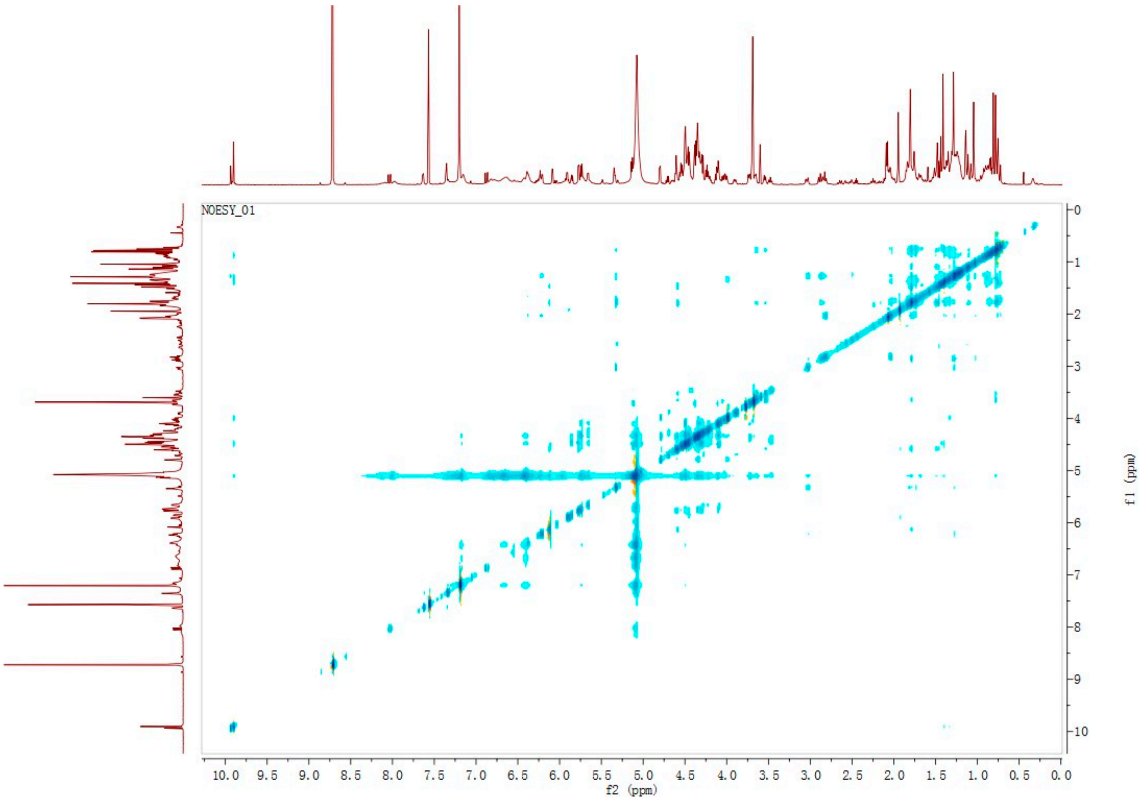

Figure S8. NOESY spectrum of compound **1**.

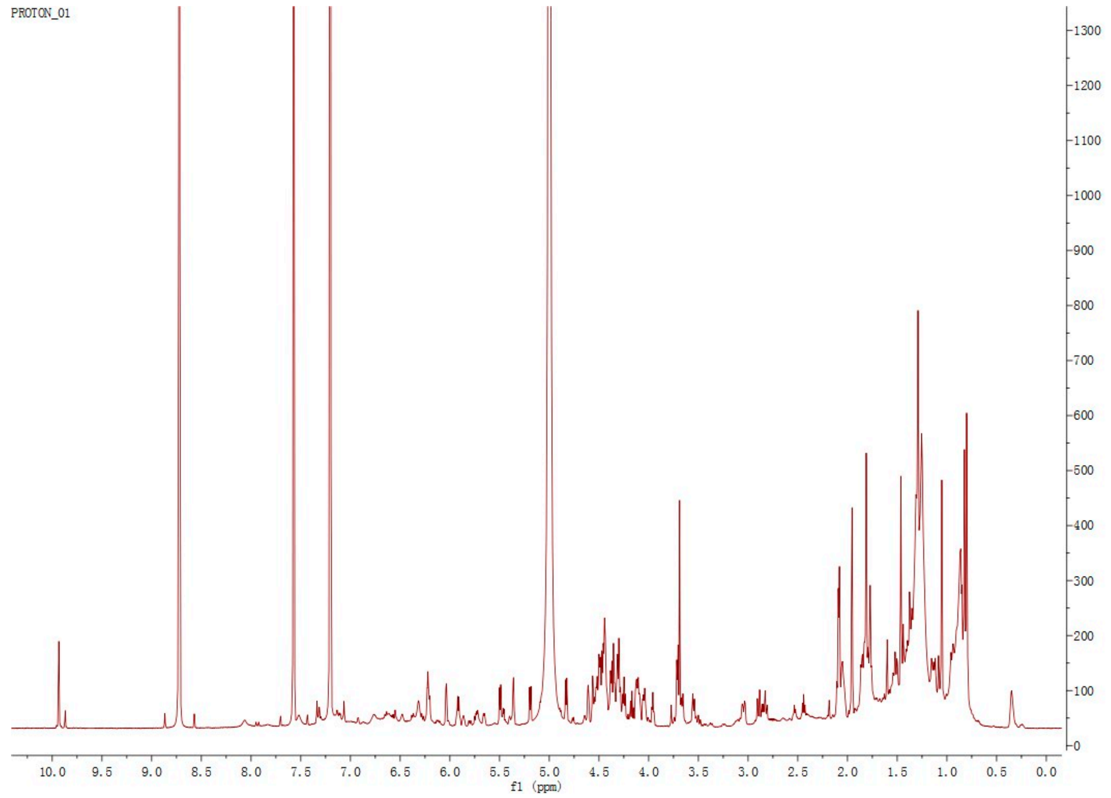

Figure S9. ^{1}H-NMR spectrum of compound **2**.

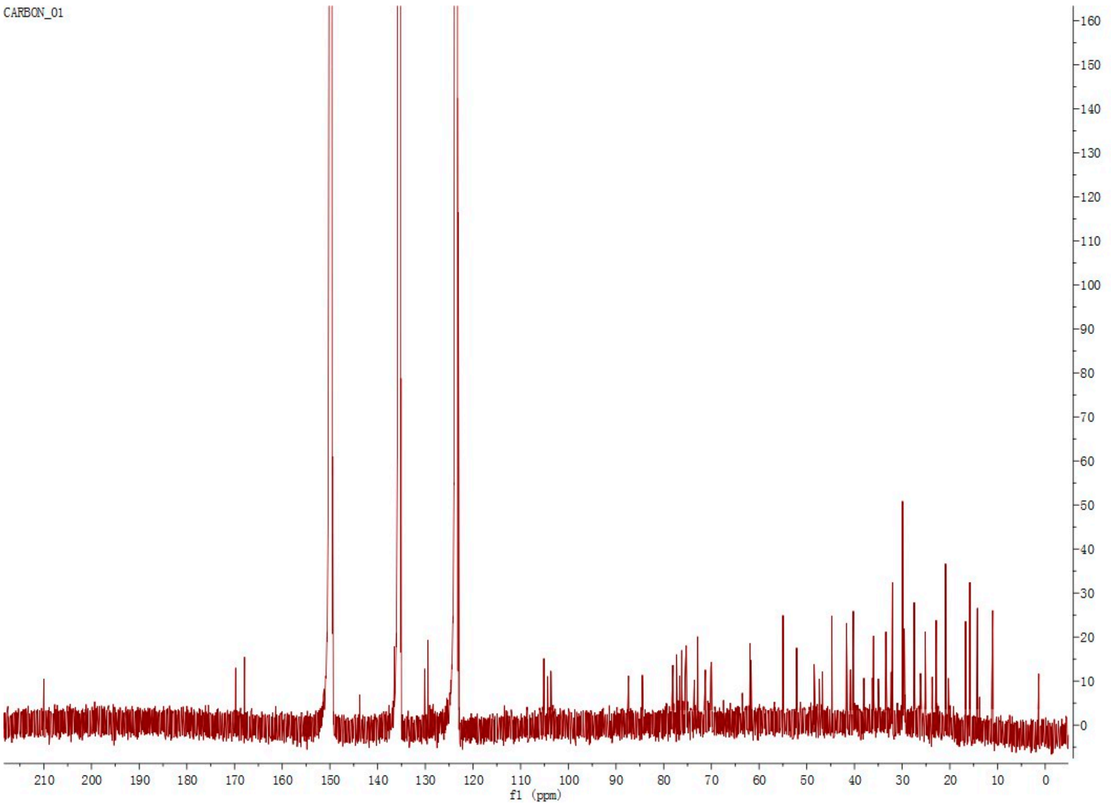

Figure S10. ^{13}C-NMR spectrum of compound **2**.

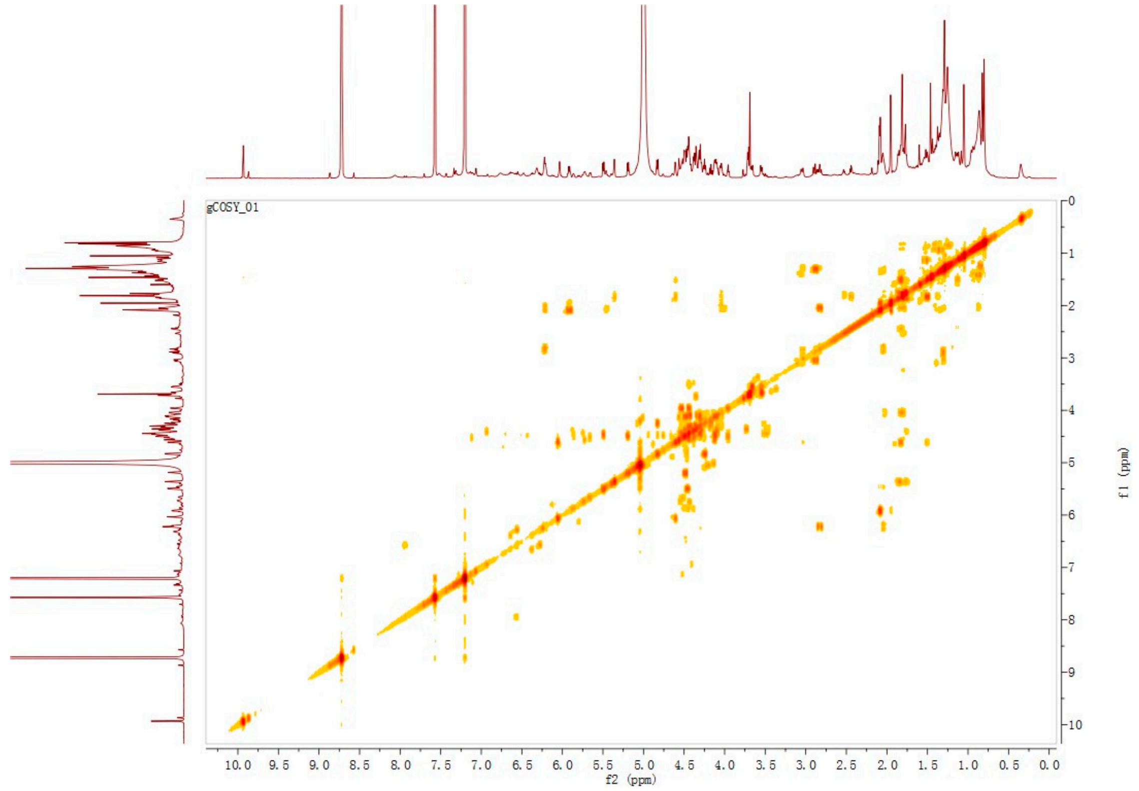

Figure S11. ^{1}H-^{1}H COSY spectrum of compound **2**.

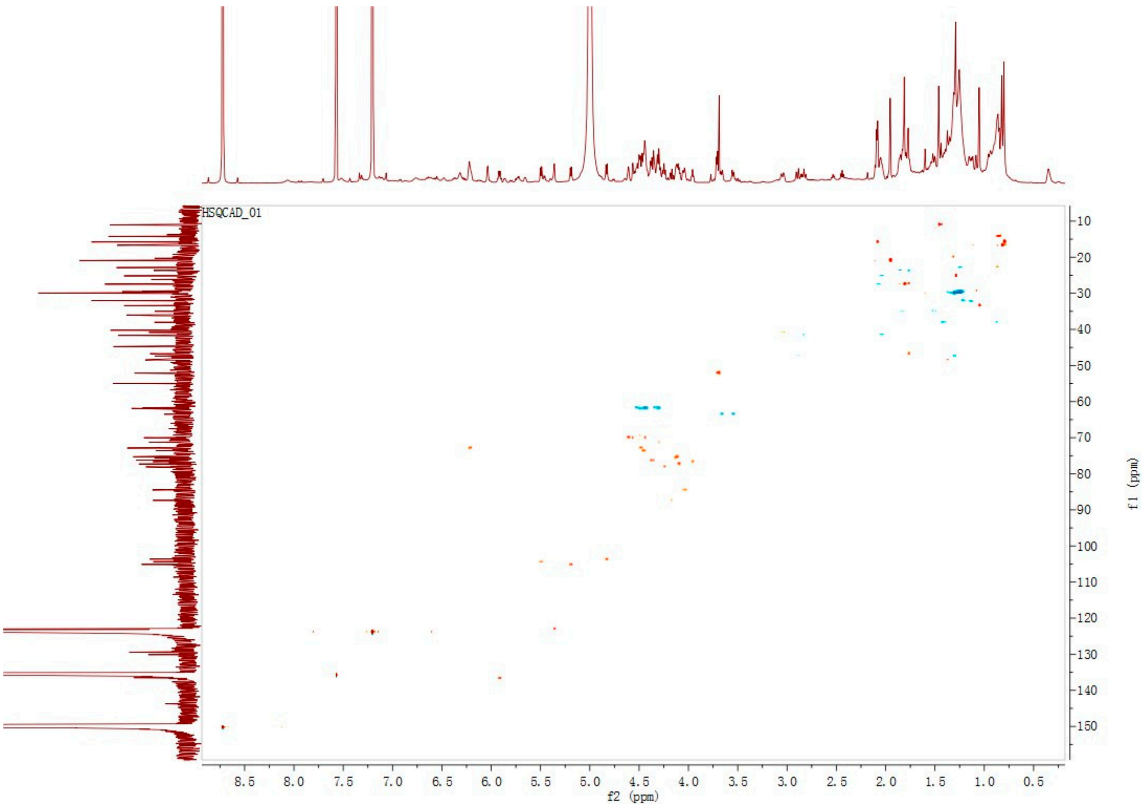

Figure S12. HSQC spectrum of compound **2**.

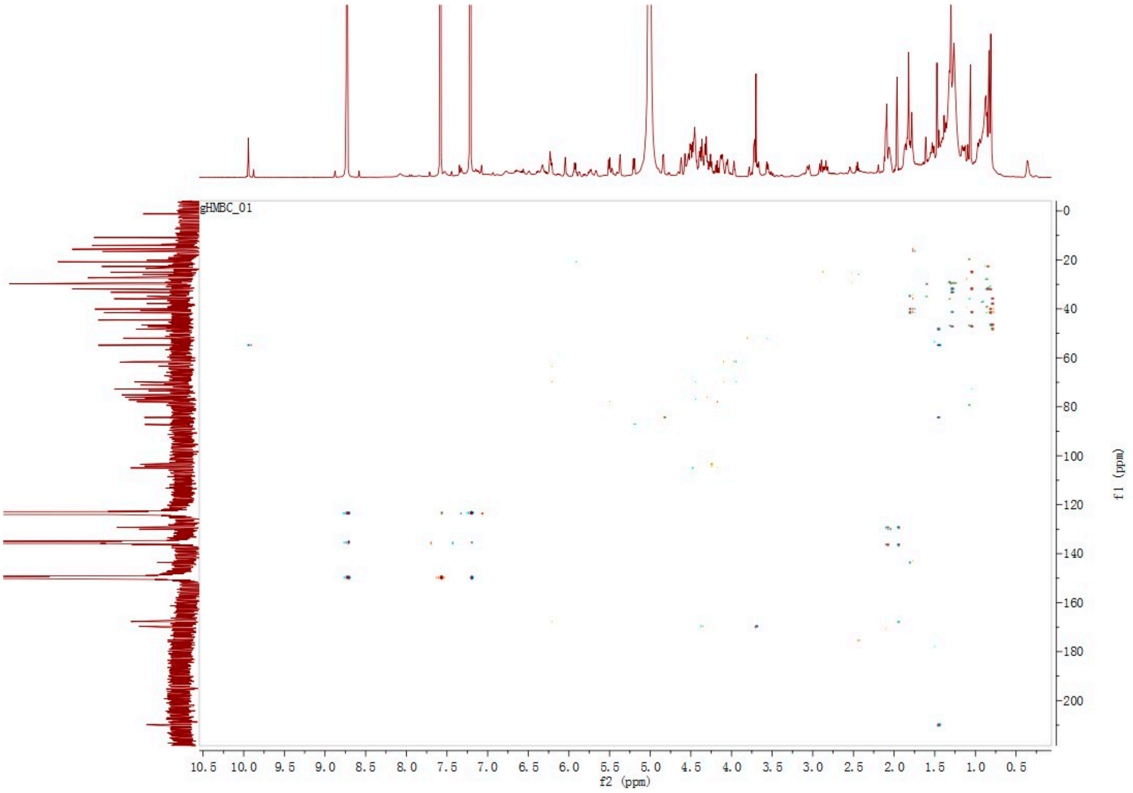

Figure S13. HMBC spectrum of compound **2**.
